# Decision-Making in Multiple Sclerosis Consultations in Italy: Third Observer and Patient Assessments

**DOI:** 10.1371/journal.pone.0060721

**Published:** 2013-04-02

**Authors:** Erika Pietrolongo, Andrea Giordano, Monica Kleinefeld, Paolo Confalonieri, Alessandra Lugaresi, Carla Tortorella, Maura Pugliatti, Davide Radice, Claudia Goss, Christoph Heesen, Alessandra Solari, the AutoMS group

**Affiliations:** 1 Department of Neuroscience and Imaging, University “G. d’Annunzio” of Chieti-Pescara, Chieti, Italy; 2 Unit of Neuroepidemiology, Foundation IRCCS Neurological Institute C. Besta, Milan, Italy; 3 Unit of Neuromuscular Diseases, Foundation IRCCS Neurological Institute C. Besta, Milan, Italy; 4 Departments of Neurological and Psychiatric Sciences, University of Bari, Bari, Italy; 5 Department of Clinical and Experimental Medicine, University of Sassari, Sassari, Italy; 6 Division of Epidemiology and Biostatistics, European Institute of Oncology, Milan, Italy; 7 Department of Public Health and Community Medicine, University of Verona, Verona, Italy; 8 Institute for Neuroimmunology and Clinical MS Research (inims), University Medical Center Hamburg-Eppendorf, Hamburg, Germany; University Hospital La Paz, Spain

## Abstract

**Objective:**

To assess decision-making in multiple sclerosis (MS) from third observer and patient perspectives.

**Method:**

Audio recordings of first-ever consultations with a participating physician (88 outpatients, 10 physicians) at four tertiary MS care clinics in Italy, were rated by a third observer using the Observing Patient Involvement in Shared Decision Making (OPTION) and by patients using the Perceived Involvement in Care Scale (PICS).

**Results:**

Mean patient age was 37.5, 66% were women, 72% had MS, and 28% had possible MS or other disease. Mean PICS subscale scores (range 0 poor, 100 best possible) were 71.9 (SD 24.3) for "physician facilitation" (PICS-F); 74.6 (SD 22.9) for "patient information exchange" (PICS-I); and only 22.5 (SD 16.2) for "patient decision making" (PICS-DM). Mean OPTION total score (0 poor, 100 best possible) was 29.6 (SD 10.3). Poorest OPTION scores were found for items assessing “preferred patient approach to receiving information” and “preferred patient level of involvement.” Highest scores were for “clinician drawing attention to identified problem”, “indicating need for decision making,” and “need to review the decision.” Consultation time, woman physician, patient-physician gender concordance and PICS-F were associated with higher OPTION total score; older physician and second opinion consultation were associated with lower OPTION score.

**Conclusions:**

In line with findings in other settings, our third observer findings indicated limited patient involvement abilities of MS physicians during first consultations. Patient perceptions of physician skills were better than third observers’, although they correlated. Consultations with women physicians, and younger physicians, were associated with higher third observer and patient-based scores. Our findings reveal a need to empower Italian MS physicians with better communication and shared decision-making skills, and show in particular that attention to MS patient preferences for reception of information and involvement in health decisions, need to be improved.

## Introduction

Patient-centered care is widely acknowledged as a core value in medicine. Originally developed in family medicine, patient-centered care has six dimensions: exploring illness experience as well as the disease, understanding the whole person, finding common ground, incorporating prevention and health promotion, enhancing the patient-physician relationship, and being realistic [1]. Shared decision making (SDM) is also a cornerstone of patient-centered care: health decisions should be made jointly by the health professional and the patient, based on the best available evidence and on patient values [2], [3]. Patient-centered care and SDM have been associated with increased patient satisfaction and empowerment, and reduced decisional conflict and treatment non-compliance [4], [5], as well as improved care provider satisfaction, strengthened patient-physician alliance, and reduced medical litigation [6]. SDM is especially important in gray-zone situations where available treatments have important risks as well as benefits, and where evidence is lacking [7], [8].

Multiple sclerosis (MS) is a chronic disease of the central nervous system that typically manifests in young adults, and affects women 2–3 times more than men. MS patients have to evaluate complex information and face difficult decisions shortly after diagnosis – at a time of heightened anxiety [9]. When deciding about disease-modifying treatments, patients need to be informed, and their perceptions of the benefits and harms of treatments must be taken into careful consideration. Sub-optimal adherence to and withdrawal from injectable first-line disease-modifying therapies are common [10]. With newer disease-modifying therapies, treatment decisions are even more complex: although more effective and easier to administer, the new drugs pose greater risks of severe side effects [11]. The situation is further complicated by the supposed association between MS and chronic cerebrospinal venous insufficiency: perhaps thousands of MS patients have undergone endovascular treatment despite the lack of evidence of efficacy [12]. In such a scenario, SDM may be crucial for preventing patients choosing options of no proven benefit [7]. Up to date, understandable evidence-based patient information is an essential part of SDM: without adequate information shared decisions are not possible [13].

The primary aim of the present study was to assess physician SDM skills in the context of MS care using a third observer scale. Secondary aims were to determine (a) the relation between patient evaluation of the consultation and third observer evaluation, and (b) characteristics associated with physician skills.

This study was part of the AutoMS project (Autonomy preferences, risk knowledge and decision making performance in MS patients; www.automsproject.org), an international initiative involving several European and one Australian center [14].

## Methods

### Participants and procedures

We recorded first-ever patient consultations with a participating physician occurring between April and December 2011 at four Italian MS centers in Northern (one research hospital), Central (one mainland and one Sardinian university hospital) and Southern Italy (one university hospital). All physicians at each MS center were eligible provided they gave written consent to participate and for the consultation to be recorded. The specific objectives and instruments of the study were not disclosed; no physician had received SDM training.

Eligible patients were age 18 years or older and able to give informed consent; those already being followed at the MS outpatient center were excluded. Patients were approached prior to the consultation by a researcher (study nurse, physician or psychologist) who presented the project, and secured consent to participate. Participating patients then self-completed the Hospital Anxiety and Depression Scale (HADS) [15], [16] and were administered the Control Preference Scale (CPS) and a socio-demographic questionnaire. After the consultation, patients rated the interview using the self-completed Perceived Involvement in Care Scale (PICS). The physician completed the patient case record form.

The consultations were unobtrusively audio-taped and transcribed verbatim; subsequently they were rated by a third observer using the Observing Patient Involvement in Shared Decision Making (OPTION) scale.

### Ethics statement

All the study patients and physicians gave written consent to participate and for the consultation to be recorded. The protocol was approved by the Ethics Committee of the following hospitals: Foundation IRCCS Neurological Institute C. Besta, Milan; University “G. d’Annunzio” of Chieti-Pescara, Chieti; University of Bari; University of Sassari; all in Italy.

### Instruments

OPTION (www.optioninstrument.com) is an observer-based scale that evaluates the behavior of the physician in terms of patient involvement in decision-making [17], [18]. It consists of 12 items, each rated on a five-point Likert scale ranging from 0 (behavior not observed) to 4 (behavior observed to high standard). A total score (range 0–48) is obtained by adding the scores of each item. OPTION has been validated in seven languages, including Italian [19], [20].

Three researchers (EP, MK, AS) were OPTION raters (third observers). After a day’s training with OPTION, they rated a subset of the consultations (audio recordings and transcripts) independently, and then met to compare scores and reach a common understanding of what the 12 items seek to elicit, especially those for which agreement was low, and also to add coding cues. A second subset of consultations was then evaluated to assess inter-rater agreement. Further examination of consultation subsets was contemplated if inter-rater agreement was insufficient. OPTION item and total scores were reported in the present study on a 0–100 scale, where 100 corresponds to the highest possible score (best possible patient involvement in decision-making).

PICS is a self-completed scale that assesses the patient’s perceived role in the consultation [21]. It consists of 13 items (each answered “yes“ or “no“) grouped into three sub-scales assessing physician facilitation of patient involvement (PICS-F, items 1–5), patient information exchange (PICS-I, items 6–9), and patient participation in decision making (PICS-DM, items 10–13).

We modified the scale by replacing yes/no answers with 4-point Likert scale preferences, ranging from “1” (completely) to “4” (not at all). The three PICS subscale scores were obtained by adding the scores of each item. Item and total scores were reported in the present study on a 0–100 scale, where 100 corresponds to the highest possible perceived involvement in the consultation.

As part of the AutoMS project, PICS was translated and culturally adapted from the original US English into Italian, Dutch for Belgium, French, Estonian and Serbian, following accepted guidelines [22], [23]. The Italian translation was a three phase process. In phase 1, two qualified translators, one an Italian native speaker and proficient in English, the other an English native speaker, both living in Italy, produced two independent forward translations. A panel consisting of the translators, a physician, a psychologist, and a lay person reviewed the forward translations and a consensus version was arrived at. In phase 2, the consensus translation generated in phase 1 was independently translated back into English by a third qualified translator, an Italian native speaker proficient in English and living in Italy. The backward translation was produced without access to the original PICS and without consulting the other translators. In a phase 3 meeting between those participating in phase 1 and the backward translator, the backward translation was compared with the original, and further refinements to the Italian version were made. Differences were resolved by discussion.

CPS was developed to evaluate the preference of an individual regarding his/her involvement in health decisions [24]. It consists of five cards, each illustrating a different role in decision-making by means of a cartoon and a short descriptive statement: the examiner asks the subject to choose the preferred card, which is then covered up; the examiner then asks the subject to choose the preferred card from the remaining four cards. The procedure continues (four choices) until one card is left. If the second preference is incongruent with the first (non adjacent pairing, such as card A with card C) the test is explained again, and re-administered. In the event of further incongruences the test is abandoned. Six scores are possible based on the person's two most preferred roles, these are collapsed to: “active” (active–active or active–collaborative), “collaborative” (collaborative–active or collaborative–passive), or “passive” (passive–collaborative or passive–passive). We used the Italian version of CPS [25], and the modified cartoons elaborated as part of AutoMS (paper in preparation).

### Statistical analysis

Categorical variables were summarized as counts and percentages and compared using the chi-squared test or Fisher’s exact test, as appropriate. Continuous variable were summarized as means and standard deviations (SD), or medians with interquartile ranges (IQR); they were compared using Kruskal-Wallis, Wilcoxon rank-sum test, or multilevel mixed-effects ANOVA with physician’s characteristics nested within center. Normality and equality of variance assumptions were tested using Shapiro-Wilk’s and Bartlett’s tests, respectively. Internal consistency was assessed by Cronbach’s alpha, with values above 0.70 considered acceptable [26]. Correlations were assessed with Spearman’s rho. Inter-rater reliability was assessed with the intraclass correlation coefficient (ICC) [27] with 95% confidence intervals (CI) determined using the bootstrap method (5000 replications).

Analyses were performed with the Stata Statistical Software, release 12 (Stata, College Station, Texas, USA). All statistical tests were two-tailed; differences were considered significant at an alpha level of <0.05.

## Results

### Participants

Of the 117 patients approached, 25 refused (21%). Lack of time was the most frequent reason given for refusal. Four audio recordings were incomplete and could not be rated. Thus, 88 consultations were analyzed. The mean age of these patients was 37.5 years; 58 (66%) were women and 63 (72%) had MS or clinically isolated syndrome (CIS). The remaining participants had suspected MS, radiologic isolated syndrome, optic neuritis or other diagnoses (Table 1). One patient (diagnosis: headache) asked for a copy of the audio-recording.

**Table 1:** Characteristics of the 88 patients participating in the study.

Characteristic	Sub-characteristic	Number of patients (%) unless otherwise indicated
Women		58 (66)
Age (years)^a^		37.5, 11.4 (20–69)
Diagnosis	MS/CIS	63 (72)
	Other condition^b^	25 (28)
Index problem: second opinion		22 (25)
Highest level of education (years)	Primary (5–8)	23 (26)
	Secondary (12–13)	48 (55)
	College/University (14+)	17 (19)
Current employment status	Employed, full-time	46 (53)
	Employed, part-time	12 (14)
	Homemaker	11 (13)
	Student	8 (9)
	Unemployed	6 (7)
	Retired (age)	3 (3)
	Disability pension	1 (1)
Disease course (n = 63 MS/CIS patients)	First episode/relapsing-remitting	56 (89)
	Relapsing-progressive/chronic progressive	7 (12)
EDSS score^c^ (n = 63 MS/CIS patients)		2.0 (1.5–3.5)
HADS (n = 87 patients with valid scores)^a^	Anxiety	7.8, 4.0 (0–19)
	Depression	4.5, 3.5 (0–14)
PICS subscale scores^a^	Physician facilitation (PICS-F)	71.9, 24.3 (7–100)
	Patient information exchange (PICS-I)	74.6, 22.9 (17–100)
	Patient decision making (PICS-DM)	22.5, 16.2 (0–67)
CPS role preference (n = 82 patients with valid scores)	Active	13 (16)
	Collaborative	47 (57)
	Passive	22 (27)
OPTION total score^a^		29.6, 10.3 (10–54)

MS is multiple sclerosis, CIS is clinically isolated syndrome, EDSS is Expanded Disability Status Scale, HADS is Hospital Anxiety and Depression Scale, PICS is Perceived Involvement in the Consultation Scale, CPS is Control Preference Scale, OPTION is Observing Patient Involvement in Shared Decision Making.

a Mean, SD (minimum–maximum).

b Encepalopathy/myelopathy (n = 10); Suspected MS (n = 9); Radiologic isolated syndrome (n = 2); Optic neuritis (n = 1); Headache (n = 1); Chronic inflammatory demyelinating polyneuropathy (n = 1); Facial spasm (n = 1). The diagnosis was provided by the physician on the case report form.

c Median (interquartile range).

Mean HADS anxiety (HADS-A) score was 7.8, and mean HADS depression (HADS-D) score was 4.5; the normality assumption for score distribution was rejected for HADS-D (p = 0.01).

Six patients gave incongruent CPS answers from which it was impossible to obtain scores; CPS scores were therefore available for 82 patients (93%): of these 57% preferred a collaborative role, 27% a passive role, and 16% an active role (Table 1).

Median physician age was 47.5 years (range 30–51), and median experience with MS was 7.5 years (range 3–24). Five of the 10 physicians were women. The median number of consultations recorded by a single physician was 9 (interquartile range, IQR 2–17) (Table 2). The Milan, Chieti and Bari physicians were neurologists, and all four Sassari physicians were residents.

**Table 2:** Characteristics of participating centers (A), consultation type (B), and physicians taking part in consultations (C).

		Milan		Chieti	Bari	Sassari	
**(A)**	**Characteristics of MS center**	**Sub-characteristic**	**Number (%)**	**Number (%)**	**Number (%)**	**Number (%)**	**P value**
	No. of beds		6	2	4	6	
		Day-hospital	2 (33)	2 (100)	4 (100)	6 (100)	
	Personnel	Neurologist	2	2	6	3	
		Resident	1	3	4	8	
		Psychologist	0	1	1	1	
		Nurse	0	3	2	0	
		Secretary	1	1	0	1	
	No. of patients followed		700	1,800	3,200	900	
**(B)**	**Characteristics of the rated consultations**	**Sub-characteristic**	**Number (%)**	**Number (%)**	**Number (%)**	**Number (%)**	**P value**
	No. of consultations		34 (39)	26 (29)	20 (23)	8 (9)	
	Diagnosis	MS/CIS	19 (56)	18 (69)	18 (90)	8 (100)	0.01
		Other conditions	15 (44)	8 (31)	2 (10)	0 (0)	
	Second opinion		15 (44)	5 (19)	2 (10)	0 (0)	0.008
	Consultation time (min)^a^		35.3 (8.6); 34.5 (20–56)	53.7 (18.4); 56.5 (19–101)	44.9 (17.1); 44.5 (23–94)	29.5 (15.2); 22.5 (14–53)	<0.001
**(C)**	**Characteristics of the 10 physicians**						
	Men/women	2/0		0/2	1/1	2/2	
	Age (years)^b^	50 (49–51)		50 (44–50)	44 (44–44)	30 (30–30)	
	MS experience (years)^b^	6 (3–10)		24 (18–24)	20 (18–20)	7 (6–7)	
	No. of consultations^b^	17 (17–17)		13 (9–17)	10 (9–11)	1 (1–4)	

MS is multiple sclerosis; CIS is clinically isolated syndrome.

a Mean (SD); Median (range).

b Median (interquartile range).

### Inter-observer reliability of OPTION ratings

The first 15 consultations (audio recordings and transcripts) available for evaluation were rated independently by the three raters (EP, MK, AS). Inter-rater reliability for OPTION total score ranged from moderate (ICC 0.64, 95% CI 0.13–0.86 for MK vs. AS) to substantial (ICC 0.90, 95% CI 0.60–0.92 for EP vs. AS). In 11 instances inter-rater disagreement exceeded one point at the item level, and in 4 instances exceeded 3 points at the total score level. Subsequently, the raters met to examine the consultations and achieve consensus on discordant item ratings. The second set of 14 consultations was rated independently: reliability improved slightly (ICC ranging from 0.70, 95% CI 0.15–0.90 for MK vs. AS to 0.90, 95% CI 0.67–0.96 for EP vs. AS); moreover, in one case only inter-rater disagreement exceeded one point at the item level, and in no instance did it exceeded 3 points at the total score level. The remaining 59 consultations were assigned to one of the three raters (EP 21, MK and AS 19 each).

### Italian version of PICS

The various phases of PICS translation-adaptation are illustrated in Table S1. No major difficulties were encountered during this process. Minor changes were made to item replies and the introduction. Two sentences were added to the introductory statement to improve clarity and provide context: The original had, “Please mark the box which is most applicable to the following statements;” while the Italian version has “PICS aims to assess your experience of the consultation of [day / month / year]. Please tick, for each statement, the box that best describes your experience. Please do not skip any of the 13 statements”.

Replies: A four-point verbal Likert scale was considered a better way of grading the replies than the original Yes/No. Scale replies can be collapsed to Yes (first two replies) or No (last two replies) to conserve comparability with other studies.

All patients completed the PICS without skipping any item. Scores were skewed to high values for PICS-F (20% of patients scored 100) and PICS-I (23% of patients scored 100), and to low values for PICS-DM (22% of patients scored 0) (Tables 1 and 3). The normality assumption was not rejected for PICS-DM (p = 0.73), and p values were 0.06 for PICS-F and PICS-I. We nevertheless use non-parametric statistical tests. Correlations between item score and total score were acceptable for PICS-F (range 0.63–0.86) and PICS-I (range 0.71–0.85), and borderline for PICS-DM (range 0.58–0.67). Scale internal consistency was acceptable or better for PICS-F (Cronbach alpha 0.80) and PICS-I (0.79), and was below reference for PICS-DM (0.47) [26].

**Table 3:** Distribution of standardized scores on each item of the Perceived Involvement in the Consultation Scale (PICS) for the 88 consultations.

Subscale	Item no. and description	Median (IQR)
PICS-F	1. My doctor asked me whether I agree with his/her decisions	100 (67–100)
	2. My doctor gave me a complete explanation of my medical symptoms or treatment	100 (100–100)
	3. My doctor asked me what I believe is causing my medical symptoms	67 (33–100)
	4. My doctor encouraged me to talk about personal concerns related to my medical symptoms	100 (67–100)
	5. My doctor encouraged me to give my opinion about my medical treatment	67 (33–100)
PICS-I	6. I asked my doctor to explain the treatment or procedure to me in greater detail	100 (67–100)
	7. I asked my doctor for recommendations about my medical symptoms	83 (67–100)
	8. I went into great detail about my medical symptoms	67 (67–100)
	9. I asked my doctor a lot of questions about my medical symptoms	67 (67–100)
PICS-DM	10. I suggested a certain kind of medical treatment to my doctor	0 (0–0)
	11. I insisted on a particular kind of test or treatment for my symptoms	0 (0–0)
	12. I expressed doubt about the test or treatment that my doctor recommended	0 (0–17)
	13. I gave my opinion [agreement or disagreement] about the type of test or treatment that my doctor ordered	33 (0–100)

Items 1–5 pertain to the physician facilitation subscale (PICS-F), items 6–9 to the patient information exchange subscale (PICS-I), and items 10–13 to the patient decision-making subscale (PICS-DM). IQR is interquartile range.

### Patient and observer views of the consultation

Patient scores were high (high perceived involvement) for PICS-I (median 75, IQR 58–92) and PICS-F (median 73, IQR 53–93), but low for PICS-DM (median 25, IQR 8–33). The distribution of scores for individual PICS items is shown in Table 3: median score ranged from 67% to 100% for the five PICS-F and the four PICS-I items. By contrast, three of the four PICS-DM item scores had a median value of zero, while one (item 13, “I gave my opinion [agreement or disagreement] about the type of test or treatment that my doctor ordered”) had a median score of 33%.

**Figure 1:**
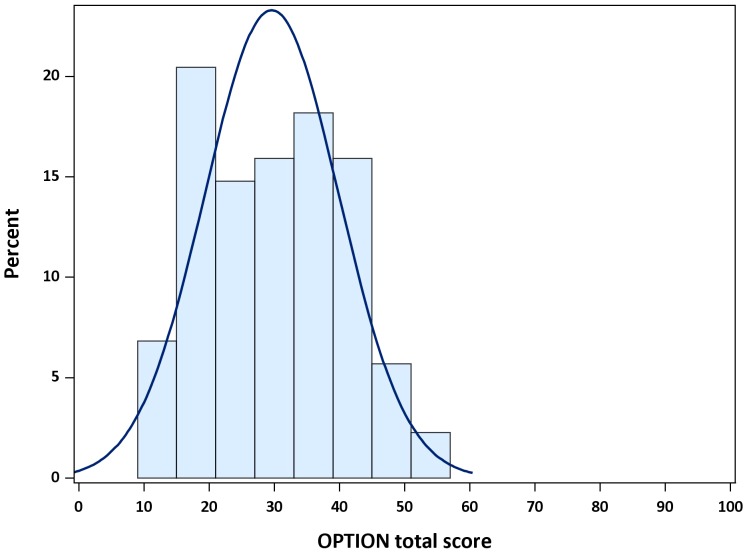
Frequency distribution of Observing Patient Involvement in Shared Decision Making (OPTION) total score (88 consultations). The line is the normal density plot. The x axis shows the full range of possible scores (0–100).

Mean OPTION total score was 29.6 (median 27.1, IQR 20.8–37.5), corresponding to a modest degree of patient involvement (Table 1). The normality assumption was not rejected (p = 0.09), and scores were within the 10–54 range (Figure 1). With regard to individual OPTION items, physicians were least likely to assess patient's preferred approach to receiving information (item 3) and patient's preferred level of involvement (item 10) (Table 4). By contrast, they were most likely to draw attention to an identified problem requiring a decision (item 1), to verify that the patient had understood the information (item 8), to indicate the need for making a decision, and for reviewing it (items 11 and 12) (Table 4).

**Table 4:** Distribution of standardized scores on each Observing Patient Involvement in Shared Decision Making (OPTION) item for the 88 consultations.

Item no. and description	Median (IQR)
1. Drawing attention to an identified problem as one that requires a decision-making process	50 (50–75)
2. Stating that there is more than one way to deal with the identified problem (equipoise)	25 (25–50)
3. Assessing the patient’s preferred approach to receiving information to assist decision making (e.g., discussion, reading printed material, assessing graphical data, using videotape or other media)	0 (0–0)
4. Listing options, which can include the choice of “no action”	25 (25–50)
5. Explaining the pros and cons of options to the patient (taking “no action” is an option)	25 (0–50)
6. Exploring the patient’s expectations (or ideas) about how the problem(s) is to be managed	25 (0–25)
7. Exploring the patient’s concerns (fears) about how the problem(s) is to be managed	0 (0–25)
8. Checking that the patient has understood the information	50 (25–50)
9. Offering the patient explicit opportunities to ask questions during the decision-making process	25 (25–50)
10. Eliciting the patient’s preferred level of involvement in decision making	0 (0–0)
11. Indicating the need for a decision making (or deferring) stage	50 (25–50)
12. Indicating the need to review the decision (or deferment)	50 (25–75)

IQR is interquartile range.

OPTION total score correlated moderately with consultation length (rho 0.33; p = 0.002) and PICS-F (rho 0.39, p<0.001), but not with PICS-I (rho 0.04, p = 0.99) or PICS-DM (rho -0.14, p = 0.18).

By ANOVA, physician characteristics associated with OPTION total score were female gender (direct association) and age >47.5 years (inverse association). Other variables significantly and directly associated with OPTION were PICS-F score, CIS/MS diagnosis, consultation time, and patient-physician gender concordance; second opinion consultations were inversely associated with OPTION total score (Table 5). Women physicians had longer consultations (mean 48.4 min, SD 20.2) than men physicians (mean 36.7 min, SD 10.0; p =  0.003), but consultation time did not differ in gender-concordant patient-physician dyads (mean 44. 6 min, SD 18.5) compared to non-concordant dyads (mean 41.0 min, SD 15.6; p = 0.26). Women physicians had higher scores than men for all 10 OPTION items with score >0 (p<0.005 for items 1, 2, 4, 5, 7, 11, 12). Characteristics associated with high PICS-F subscale were physician gender (women median 80, IQR 67–93 vs. men 60, IQR 40–93; p = 0.04), and physician age (≤47.5 years median 87, IQR 60–93 vs. >47.5 years median 73, IQR 53–93; p = 0.04). Consultation time, and the other physician and patient variables were not associated with PICS-F.

**Table 5:** Characteristics associated with Observing Patient Involvement in Shared Decision Making (OPTION) in multilevel mixed-effects ANOVA.

	β (95% CI)	P value
**Patient characteristics**		
Women	−1.8 (−6.4–2.8)	0.45
Age (years; square-root transformed)	−2.2 (−9.6–5.1)	0.55
Education, secondary or more	−2.0 (−7.0–3.0)	0.43
MS/CIS diagnosis	**6.2 (1.5–10.8)**	**0.01**
HADS Anxiety	−0.1 (−4.5–4.3)	0.97
HADS Depression (square-root transformed)	−2.1 (−7.5–3.3)	0.44
CPS role, active/collaborative	0.4 (−4.8–5.6)	0.87
PICS-F >73.3^b^	**6.6 (2.4–10.7)**	**0.003**
PICS-I >75.0^b^	−0.0 (−4.6–4.5)	0.98
PICS-DM	−0.1 (−0.2–0.0)	0.13
**Physician characteristics**		
Women^a^	**10.0 (6.1–13.8)**	**<0.001**
Age >47.5 years^a,b^	−**10.3 (**−**14.8– -5.7)**	**<0.001**
Experience with MS >7.5 years^a,b^	−0.7 (−5.5–4.0)	0.76
**Other characteristics**		
Consultation time (min; log transformed)	**6.3 (0.9–11.7)**	**0.02**
Consultation for second opinion	−**6.5 (**−**11.1– -1.6)**	**0.009**
Patient-physician of same gender	**5.1 (0.8–9.5)**	**0.02**

MS is multiple sclerosis, CIS is clinically isolated syndrome, EDSS is Expanded Disability Status Scale, HADS is Hospital Anxiety and Depression Scale, PICS is Perceived Involvement in the Consultation Scale, CPS is Control Preference Scale, β (95% CI) is regression coefficient with 95% confidence intervals. ^a^Entered as nested effect within center. ^b^Non-normally distributed continuous variable, categorized (median value as cutoff).

## Discussion

Besides being competent in their specialty, physicians are expected to communicate effectively with their patients and engage them in decision-making. However, despite widespread endorsement of the SDM approach by the medical community, physicians seem to apply it insufficiently during consultations [2], [8], [18], [19], [28]. In a review of the SDM skills displayed by physicians of various specialties using the OPTION scale, Pellerin et al. found low skills, except in one study on physicians who had completed SDM training [8]. The mean OPTION total score in our study was only 30 of a possible 100, in line with findings of a German study assessing video-recorded consultations in a randomized controlled trial on a patient decision-aid about MS immunotherapy [28], [29]. Our data are also in line with findings in non-MS settings [18], [19], [30], [31], suggesting that SDM is not a prominent part of patient care.

Also in line with other publications, the SDM skills displayed by our physicians correlated significantly with the duration of the consultation [8], [19], [20], perhaps because a longer meeting increases the probability that the physician manifests SDM skills. However, the time allocated for a consultation is structure-dependent, and time and consultation room availability are often at a premium, particularly in the current economic climate. Furthermore, professionals given SDM training learn to incorporate SDM behaviors without increasing consultation time [32].

Women physicians had a higher OPTION score than men, in line with a previous study [33], perhaps due to a greater propensity of women to listen and communicate [34]. Other variables we found associated with higher OPTION score were young physician age, and patient-physician gender concordance. Second-opinion consultations, and diagnoses other than MS or CIS, were associated with lower OPTION scores which might be due to subconsciously reduced physician interest in SDM for patients who will not be followed personally.

The four OPTION scale SDM behaviors that our physicians displayed most often were: drawing attention to an identified problem requiring a decision; checking that the patient has understood the information; indicating the need for a decision; and indicating the need to review that decision. By contrast, in no case did physicians elicit patient's preferred approach to receiving information, or their preferred level of involvement. OPTION studies in cardiology, psychiatry and oncology have reported closely similar findings [30], [33], [35], [36], suggesting either that physicians think that they can intuit patient preferences, or that they are not comfortable asking about them, not even at the first encounter, as in this study. In this context it is noteworthy that, although 27% of our MS patients preferred a passive role on the CPS (Table 1), OPTION score did not differ according to patient role preference (mean 29.8, SD 10.6 in CPS active/collaborative vs. 29.4, SD 10.3 in CPS passive), further suggesting that physicians did not consider patient preferences as relevant, or were unable to intuit or adjust to them.

A limitation of our study is that a relatively small number of consultations was recorded, preventing multivariable modeling. Furthermore one center contributed with only eight consultations, and four of the physicians there did only one consultation each, complicating the association of physician characteristics with SDM skills [8]. However, a sensitivity analysis in which this center was removed, produced closely similar results (data not shown).

A drawback of the third observer approach is that it is resource-consuming, requiring prior and on-the-job training as well as considerable time for the rating process (listening to and reading the transcripts). Furthermore, measurement of a complex and dynamic process like medical decision-making can be influenced by factors other than physician skills. For example, patients can broach OPTION elements proactively, biasing to a low OPTION score (to assess this we are planning to evaluate patient behavior in the same consultations) [28]. Moreover, in follow-up encounters decision-making behaviors can be absent because they have already taken place [17]: it is for this reason that we only assessed first-ever consultations.

To assess patient perceptions of the medical encounter we used the self-completed PICS assessing three domains: doctor facilitation of patient involvement (PICS-F), information exchange (PICS-I), and participation in decision making (PICS-DM). The two first domains provided median scores of about 70 of a possible 100, indicating physician skills were perceived more favorably by the patient than the third observer (as assessed by OPTION). A study on psychiatrists and their patients (answering OPTION questions) produced a similar finding [35]. However, comparison of OPTION and PICS findings is not straightforward because of the construct difference. It is also possible that, even though patients were informed that their evaluation was confidential, acquiescence bias contributed to high PICS scores. Another possibility is that high PICS reflects patients’ satisfaction with being asked their opinion (immediately after the consultation). It is noteworthy that OPTION total score and PICS-F correlated moderately, in contrast to the findings of the above cited German study [28], [29] which found no correlation between physician and patient perspectives. The sub-optimal metrics of the PICS-DM found in our and in another study [35], with three out of four items with a median score of 0 (I suggested a certain kind of medical treatment to my doctor; I insisted on a particular kind of test or treatment for my symptoms; I expressed doubt about the test or treatment that my doctor recommended) point to a careful interpretation of findings from this subscale.

Communication behaviors within the patient-physician consultation have so far been assessed using third observer-based and patient-based approaches. However, at the centre of current debates about the assessment of SDM is acknowledging the complex, interdependent nature of the medical consultation: to properly understand and evaluate the consultation, it is essential to recognize in which extent the actors involved interact, and influence each other [36]. For this purpose, a single instrument (dyadic OPTION) to assess both patient and third observer views to the medical encounter (and not focusing only on physician’s skills) has been published [36]; and a “three foci” (patient, physician and third observer) approach has been recently proposed [37]. Both tools require further validation prior to their use in clinical research settings.

To our knowledge, ours is the first multicenter study to assess SDM practice on first consultation with MS outpatients; the study of Kasper et al. [28], [29] differed in that it assessed MS consultations at a single German centre in the context of the decision to start immunotherapy. Notwithstanding these differences, mean OPTION scores were closely similar in both studies, revealing a need to empower MS physicians with better communication and shared decision-making skills. The German group is now evaluating the efficacy of such training [38]. In Italy, only in the past five years have programs on doctor-patient communication skills that advocate patient participation been incorporated into some undergraduate, postgraduate, and continuous medical education courses [39]. Studies on the efficacy of training on communication skills in the Italian context have been published [14], [40]–[45] but none were specifically concerned with SDM skills in MS. Our findings emphasize that attention to MS patient preferences for reception of information and involvement in health decisions, need to be improved.

## Supporting Information

Table S1 **Outcomes of the five phases of translation-adaptation procedure for the 13 items of the Perceived Involvement in the Consultation Scale (PICS).** The original PICS items are reproduced in Table 3.
(PDF)
