# Supplementary material for: Decision-Making in Multiple Sclerosis Consultations in Italy: Third Observer and Patient Assessments
Source: PLoS One. 2013 Apr 2;8(4):e60721. doi: 10.1371/journal.pone.0060721 (PMC3614559; doi:10.1371/journal.pone.0060721)
Supplement: Table S1 — Outcomes of the five phases of translation-adaptation procedure for the 13 items of the Perceived Involvement in the Consultation Scale (PICS). The original PICS items are reproduced in Table 3. (PDF) [file pone.0060721.s001.pdf]

Table S1. Outcomes of the five phases of translation-adaptation procedure for the 13 items of the Perceived Involvement in the Consultation Scale (PICS). The original PICS items are reproduced in Table 3.

Item no.	Independent forward translation I	Independent forward translation II	Consensus Italian version	Independent backward translation	Final Italian version
1	Il (mio) medico mi ha chiesto se sono d'accordo con le sue decisioni	Il mio medico mi ha chiesto se approvo le sue decisioni	Il medico mi ha chiesto se sono d'accordo con le sue decisioni	The doctor asked me if I agreed with his/her decisions	Il medico mi ha chiesto se sono d'accordo con le sue decisioni
2	Il medico ha fornito delle spiegazioni esaurienti in merito ai miei sintomi oppure alla mia terapia	Il medico mi ha spiegato in modo esauriente la mia sintomatologia o la cura	Il medico mi ha spiegato in modo esauriente i miei sintomi e/o la terapia	The doctor explained to me my symptoms and/or therapy exhaustively	Il medico mi ha spiegato i miei sintomi e/o la terapia in modo esauriente
3	Il medico ha chiesto se ritenevo di conoscere la causa dei miei sintomi	Il medico mi ha chiesto cosa io pensi sia la causa della mia sintomatologia	Il medico mi ha chiesto quale sia, secondo me, la causa dei miei sintomi	The doctor asked me what I believed was the cause of my symptoms	Il medico mi ha chiesto quale sia, secondo me, la causa dei miei sintomi
4	Il medico mi ha incoraggiato a parlargli delle mie preoccupazioni in merito ai miei sintomi	Il medico mi ha incoraggiato a raccontare le mie preoccupazioni riguardo alla mia sintomatologia	Il medico mi ha incoraggiato/a a parlargli/le delle mie preoccupazioni riguardo ai miei sintomi	The doctor encouraged me to talk about my concerns about my symptoms	Il medico mi ha incoraggiato/a a parlare delle mie preoccupazioni riguardo ai miei sintomi
5	Il medico mi ha incoraggiato a esprimere il mio parere in merito alla mia terapia	Il medico mi ha incoraggiato ad esprimere la mia opinione sulla terapia che seguirò	Il medico mi ha incoraggiato/a ad esprimere il mio parere riguardo alla mia terapia	The doctor encouraged me to express my opinion about my therapy	Il medico mi ha incoraggiato/a ad esprimere il mio parere riguardo alla mia terapia
6	Ho chiesto al medico di fornirmi spiegazioni più esaurienti in merito alla terapia o all'intervento	Ho chiesto al medico spiegazioni più precise sulla cura o sul percorso (terapeutico) da seguire	Ho chiesto al medico spiegazioni più dettagliate sulla terapia o procedura	I asked the doctor for more details on the therapy or procedure	Ho chiesto al medico spiegazioni più dettagliate sulla terapia e/o sugli esami

		(sulle modalità)			
7	Ho chiesto al medico dei consigli in merito ai miei sintomi	Ho chiesto al medico consigli per la mia sintomatologia	Ho chiesto consigli al medico riguardo ai miei sintomi	I asked the doctor for advice about my symptoms	Ho chiesto consigli al medico riguardo ai miei sintomi
8	Ho descritto molto accuratamente i miei sintomi	Ho descritto ampiamente la mia sintomatologia (con precisione)	Ho descritto molto dettagliatamente i miei sintomi	I described my symptoms very much in detail	Ho descritto molto dettagliatamente i miei sintomi
9	Ho rivolto al medico molte domande in merito ai miei sintomi	Ho fatto al medico molte domande sulla mia sintomatologia	Ho posto molte domande al medico riguardo ai miei sintomi	I asked the doctor many questions about my symptoms	Ho posto molte domande al medico riguardo ai miei sintomi
10	Ho suggerito al medico una determinata terapia medica	Ho suggerito al medico una determinata terapia	Ho suggerito al medico una determinata terapia	I suggested a certain type of therapy to the doctor	Ho suggerito al medico una determinata terapia
11	Ho insistito per sottopormi a un determinato esame o per seguire una determinata terapia in relazione ai miei sintomi	Ho insistito per ottenere una analisi o una terapia specifica per i miei sintomi	Ho insistito su un particolare esame o terapia per i miei sintomi	I insisted on a specific test or therapy for my symptoms	Ho insistito su un particolare tipo di esame e/o terapia per i miei sintomi
12	Ho espresso delle incertezze in merito alle terapie o agli esami proposti dal medico	Ho manifestato i miei dubbi sulle analisi o cure consigliate dal medico	Ho espresso dei dubbi sugli esami e/o terapie proposti dal medico	I expressed doubts on the tests and/or therapies suggested by the doctor	Ho espresso dei dubbi sugli esami e/o terapie proposti dal medico
13	Ho dato il mio parere (positivo o negativo) sulle terapie o sugli esami prescritti dal medico	Ho espresso la mia opinione (consenso o dissenso) sul tipo di analisi o cure prescritti dal medico	Ho dato il mio parere (favorevole o sfavorevole) sul tipo di esami e/o terapie prescritti dal medico	I gave an opinion (positive or negative) on the types of tests and/or therapies the doctor prescribed	Ho dato il mio parere (favorevole o sfavorevole) sul tipo di esami e/o terapie prescritti dal medico
